# Supplementary material for: The Polyamine Analogue Ivospemin Increases Chemotherapeutic Efficacy in Murine Ovarian Cancer
Source: Biomedicines. 2024 May 23;12(6):1157. doi: 10.3390/biomedicines12061157 (PMC11200633; doi:10.3390/biomedicines12061157)
Supplement: Supplementary file 1 [file biomedicines-12-01157-s001.zip › biomedicines-2878780-supplementary.pdf]

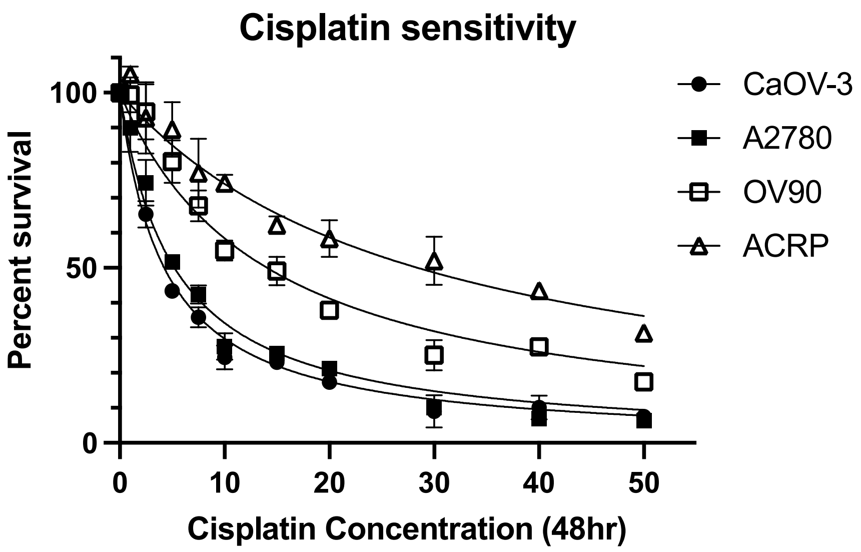

Supplementary Figure S1. Cisplatin sensitivity of human ovarian adenocarcinoma cell lines. Four human adenocarcinoma cell lines were treated with cisplatin concentrations ranging from 1μM to 50μM for 48 hours. Cell viability was determined by the Cell Titer Blue assay. The cisplatin IC50 values for each cell line are listed in Table 1.

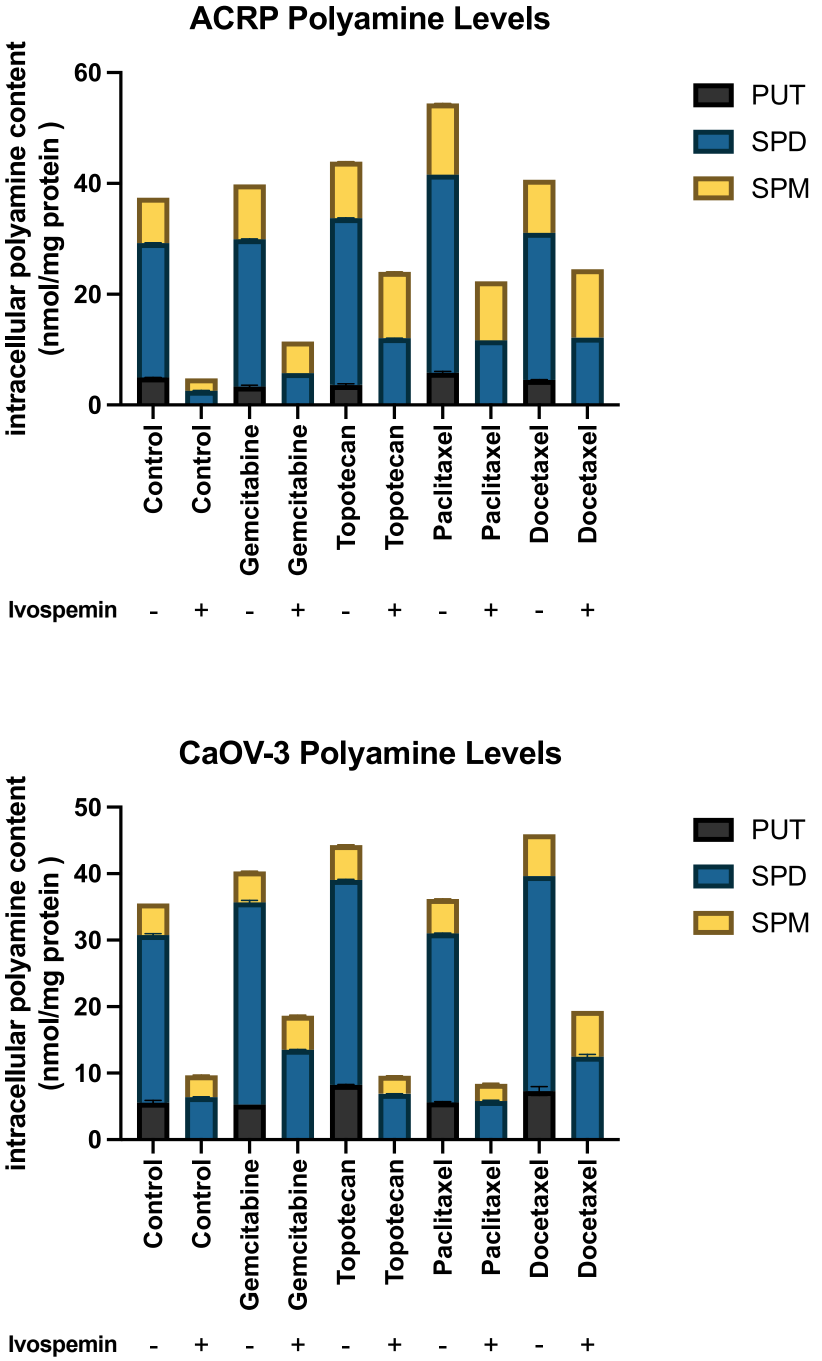

Supplementary Figure S2. Ivospemin treatment depletes polyamines independent of chemotherapy treatment. Two human ovarian adenocarcinoma cell lines were treated with 5μM ivospemin for 48 hours alone or in addition to 24 hours of chemotherapy treatment. Intracellular polyamine content was then measured from collected cells. Ivospemin reduces overall polyamine content, while chemotherapy does not appear to impact polyamine levels supporting the additivity of chemotherapeutic and ivospemin mechanisms.
